# Relationship Between Depression and Neurodegeneration: Risk Factor, Prodrome, Consequence, or Something Else? A Scoping Review

**DOI:** 10.3390/biomedicines13051023

**Published:** 2025-04-23

**Authors:** Dario Papa, Alessandro Ingenito, Alessandro von Gal, Maria Francesca De Pandis, Laura Piccardi

**Affiliations:** 1 Department of Psychology, Sapienza University of Rome, 00185 Rome, Italy; papa.2004191@studenti.uniroma1.it (D.P.); alessandro.vongal@uniroma1.it (A.v.G.); 2 International School of Advanced Studies, University of Camerino, 62032 Camerino, Italy; alessandro.ingenito@unicam.it; 3 Molecular Mind Laboratory (MoMiLab), Sensorimotor Experiences and Mental Representations (SEMper) Group, IMT School for Advanced Studies Lucca, 55100 Lucca, Italy; 4 Cassino San Raffaele Hospital, 03043 Cassino, Italy; maria.depandis@sanraffaele.it

**Keywords:** depression, neurodegeneration, risk factor, prodrome, Alzheimer’s disease, Parkinson’s disease, comorbidity

## Abstract

**Background**: The link between depression and neurodegeneration is complex and unclear. It is debated whether depression is a risk factor, a prodrome, a consequence, or unrelated. **Objectives**: This review examines these possibilities to clarify their connection, focusing primarily on Alzheimer’s disease, vascular dementia, Parkinson’s disease, and other highly comorbid neurodegenerative diseases. **Methods**: Eligibility criteria: The studies included in this review focused on neurodegenerative diseases with high comorbidity with depression, published in peer-reviewed English-language journals, providing empirical evidence on the link between the two conditions or theoretical frameworks that point to other studies. Non-human studies and those irrelevant to this connection were excluded. Source of evidence: AI-supported tools identified relevant articles. **Results**: Most studies suggest depression may contribute to neurodegeneration, but clinical, neuroimaging, and longitudinal evidence also support its role as a prodrome or consequence, indicating a bidirectional relationship. **Conclusions**: Despite extensive research, the connection remains unclear, highlighting the need for further investigation into underlying mechanisms and interdependencies, focusing on longitudinal studies by examining causality.

## 1. Introduction

Depression and neurodegeneration are two complex and prevalent conditions. Studies on the psychopathology of dementia disorders indicate that about half of patients with dementia exhibit depressive symptoms [1,2]. For this reason, in recent years, interest in the relationship between these two conditions has significantly increased, with a heated debate on the nature of this link.

Since it is now well-known that depression and dementia share a similar spectrum of cognitive symptoms [3], this scoping review aims to explore the various hypotheses attempting to explain the relationship between these two conditions.

There is considerable debate regarding which forms of neurodegeneration have the highest rates of comorbidity with depression.

This study will concentrate exclusively on neurodegenerative diseases that are primarily sporadic rather than genetically inherited. This focus is important as sporadic cases account for the majority of instances in the general population.

This makes the results highly relevant and applicable to a broader context. Moreover, focusing on these conditions helps isolate depression’s role as a risk factor, prodrome, or consequence, reducing the confusion related to genetic variability and highlighting the importance of environmental factors and lifestyle, which are crucial for public health interventions and preventive strategies. However, it should not be ignored that, albeit minimally, some of the diseases investigated may manifest in the so-called “familial” forms, and the inability to definitively classify these conditions may confound results. In this regard, while our focus on sporadic forms is justified by their higher prevalence and relevance to public health interventions, we acknowledge certain limitations of this approach. The exclusion of familial forms of neurodegenerative diseases potentially limits our understanding of the full spectrum of depression–neurodegeneration relationships. Familial forms, despite their rarity, can provide valuable insights into underlying biological mechanisms through their more clearly defined genetic pathways. These genetic variants might interact with depression in distinct ways that could illuminate mechanisms relevant to sporadic forms as well. Additionally, by excluding familial cases, we might miss important gene–environment interactions where depressive symptoms could modify the expression or progression of genetically determined neurodegenerative processes. Future research could benefit from comparative studies examining how depression interacts with both sporadic and familial forms of neurodegeneration, potentially revealing shared pathways or highlighting important distinctions in these relationships.

An interesting review by [4] explored the intricate relationship between major neurodegenerative disorders and depression. Among these, a substantial body of research suggests that “Alzheimer’s disease (AD)” and “vascular dementia (VaD)” emerged as the primary conditions linked to this emotional affliction. These two forms are the most prevalent types of dementia among the elderly, albeit with some variability, some studies report alarming depression rates as high as 87% within these populations [5].

“Parkinson’s disease (PD)” follows, with comorbidity rates found to be up to around 50% [4,6,7], along with other atypical parkinsonisms, including “dementia with Lewy bodies (DLB)”, with numerous studies showing comorbidity rates even higher than those of AD [8,9,10,11]; “corticobasal degeneration (CBD)”, and “progressive supranuclear palsy (PSP)” [12], although these latter two conditions have been less thoroughly investigated.

Additionally, “frontotemporal dementia (FTD)” is also frequently associated with depressive comorbidity [13,14], as is “amyotrophic lateral sclerosis (ALS)” [15,16,17,18].

Lastly, it would be appropriate to add “mild cognitive impairment (MCI)” to the previous list if we considered it as a transitional “pre-dementia” state, particularly of AD, as suggested by several studies [19,20,21], and considering that more than half of individuals with the disorder progress to a form of dementia within 5 years of diagnosis [22,23,24]. Depression appears to be a prevalent issue in this context as well [25,26], with approximately one-third of patients demonstrating this dual diagnosis on average [27,28].

As [29] suggests, the relationship between depression and neurodegeneration is bidirectional, complex, and multifactorial. Although research has already demonstrated a strong correlation between these two conditions, many questions remain unanswered regarding the nature of this link. To date, a minimum of four hypotheses have been proposed to explain the relationship between depression and neurodegeneration, with varying degrees of prominence: (1) depression as a risk factor or potential catalyst for neurodegeneration; (2) depression serving as an early indicator of neurodegenerative processes; (3) depression emerging as a consequence of neurodegenerative conditions; (4) depression and neurodegeneration being unrelated or spuriously related conditions. Each hypothesis presents a distinct yet potentially overlapping perspective on the association between depression and neurodegeneration, reflecting the complexity of this clinical relationship. Before examining the evidence supporting each hypothesis, a few anticipations are necessary.

1. The first thesis states that depression severely impacts the quality of life and can lead to chronic stress from neurobiological changes, increasing the risk of neurodegenerative diseases and cognitive decline [30,31].
2. Investigating the first hypothesis is challenging, as another line of research suggests that depression may be more of a prodrome—a typical symptom that precedes the actual disease, serving as a “premonitory symptom” [32,33,34]. This suggests that early-diagnosed depression may predispose to pathological aging, while “late-life depression (LLD)” could serve as a prodrome for more severe latent conditions, supporting the thesis of a dichotomous relationship between early-life depression as a risk factor and LLD as a prodrome of dementia [34,35,36,37]. While it is methodologically possible to differentiate between early and later-life depression, studies have established arbitrary cut-off points since the timing of depressive symptoms relative to neurodegenerative disease onset cannot be objectively determined [32]. Two arguments appear to overlap and could both be valid [38].
3. In parallel, other studies suggest neurodegeneration may be a key cause of depression, impacting emotional management circuits. While the psychological effects of a grim diagnosis are acknowledged, the role of neurodegeneration in depression should be emphasized [39].
4. Finally, it cannot be ruled out that the association may depend on latent variables that covary with both; hence, it is well-known that these syndromes tend to manifest comorbidly due to medical factors and therefore, with each other. As reported above, the coexistence of neurodegeneration and depression is a phenomenon that has sparked various hypotheses. To provide a comprehensive understanding of this issue, this scoping review meticulously explores the four key theories that link these two pathological conditions, aiming to shed some light on this complex relationship.

However, it remains important to emphasize up front that this scoping review represents a preliminary exploration of the relationship between neurodegeneration and depression, with the primary goal of mapping the current state of the literature and identifying key patterns, trends, and knowledge gaps. This study was not designed to provide definitive answers or causal conclusions, but rather to serve as a foundational step for future, more targeted investigations. Using a broad and flexible approach, this review aimed to capture a broad range of evidence across interdisciplinary domains. The insights gained from this scoping review may inform future research efforts by providing a clearer direction for systematic studies and meta-analyses.

## 2. Materials and Methods

This scoping review explores the relationship between neurodegeneration and depression, utilizing a systematic yet flexible framework to map and synthesize the available evidence. This scoping review was completed based on the 2018 “PRISMA guidelines for scoping reviews (PRISMA-ScR)” [40].

### 2.1. Inclusion and Exclusion Criteria

Several inclusion criteria were considered:

(i) studies discussing neurodegeneration with a high comorbidity with depressive disorders; (ii) peer-reviewed studies; (iii) only publications in English to ensure a homogeneous understanding of the existing literature; (iv) studies addressing the interaction between the two conditions, examining underlying biological mechanisms, risk factors, and clinical impacts; (v) studies on human samples.

Regarding exclusion criteria, all irrelevant studies on the interaction between neurodegeneration and depression were excluded, as well as studies discussing neurodegenerative pathologies without depressive comorbidities (and vice versa). Additionally, studies on non-human samples were excluded. No temporal restrictions were applied to the publication years of eligible entries, allowing for a broader temporal scope that could also provide insights into how the study of the relationship between these two conditions has evolved over time.

### 2.2. Preselection Phase

The process began with a preliminary investigation using “Elicit: The AI Research Assistant” [41], an AI-based search engine that uses semantic similarity algorithms to identify scientific literature relevant to a specific query. Unlike traditional search engines that rely strictly on keyword matching, Elicit can find conceptually related articles even when they do not contain the exact search terms, allowing for a more comprehensive exploration of the scientific literature on depression and neurodegeneration. Specifically, this AI-assisted approach was chosen over traditional database searches (e.g., PubMed, Scopus) for several methodological advantages: First, unlike conventional keyword searches that may miss conceptually related studies using different terminology, Elicit’s semantic analysis can identify relevant articles even when they do not contain the exact search terms. Second, given the interdisciplinary nature of our research question spanning psychiatry, neurology, and cognitive science, Elicit’s ability to synthesize literature across multiple domains provided a more comprehensive initial dataset. Third, traditional search strategies often require multiple complex Boolean queries to capture the nuanced relationship between depression and various neurodegenerative conditions, potentially missing important connections. Elicit’s natural language processing capabilities allowed us to directly ask our research questions and obtain more conceptually coherent results. Finally, the AI tool’s ranking algorithm, which considers both relevance and scientific impact, helped prioritize seminal and influential studies for our initial exploration.

The first step involved posing the following query to the aforementioned AI tool: “What are the neurodegenerative conditions with the highest comorbidity rates with depression?”.

This initial query was part of an iterative refinement process. We first examined the preliminary results, noting which neurodegenerative conditions consistently appeared with high depression comorbidity. We then conducted several follow-up queries with variations in terminology (e.g., “depressive symptoms” vs. “depression”, “neurodegenerative disorders” vs. “neurodegenerative diseases”) to ensure comprehensive coverage. The results were compared across these iterative searches to identify consistent patterns and eliminate potential terminology biases. Additionally, we refined our approach by examining the abstracts of the top-ranked articles from each iteration, which helped us identify the most relevant terminological frameworks used in the field. This progressive refinement allowed us to optimize our final query to capture the conditions with truly significant depressive comorbidity rather than those merely appearing frequently in the literature due to terminology overlap or publication volume biases.

This allowed us to identify that, among the numerous neurodegenerative conditions, only a few exhibited considerable depressive comorbidity rates and consistently appeared in the results.

The conditions that predominantly emerged included AD, VaD, PD, FTD, DLB, CBD, PSP, ALS, and MCI, which were characterized by particularly frequent comorbidity, albeit to varying degrees.

The results of this preliminary phase laid the foundation for the introductory discussion, where these conditions were contextualized within the general framework of this review.

### 2.3. Research Questions and Strategy

Four research questions guided the review:

- “Can depression be a cause of neurodegeneration?”;
- “Can depression be a prodrome or early indicator of neurodegeneration?”;
- “Can depression be a consequence of neurodegeneration?”;
- “Is there a spurious or non-existent correlation between depression and neurodegeneration?”.

These four research questions were directly input into Elicit as natural language queries using precisely this phrasing. We deliberately avoided using complex Boolean operators or specialized search terms to leverage Elicit’s semantic search capabilities. This approach allowed us to cast a wider net and capture conceptually relevant studies regardless of the specific terminology used by authors to describe these relationships. We maintained consistent language across queries to ensure a balanced representation of each hypothesis in the initial results. Additionally, each query was examined individually rather than combined to prevent any one hypothesis from dominating the search results.

To address these questions, the “most relevant” filter of Elicit was used, retrieving the top 50 articles for each query. The selection of 50 articles per query represented a deliberate methodological choice based on preliminary testing. We found that increasing the number beyond 50 led to diminishing returns in terms of relevance and thematic diversity while introducing significantly more noise into the dataset. Conversely, selecting fewer than 50 risked omitting relevant articles, particularly those exploring novel or less mainstream perspectives. This threshold allowed us to maintain a balance between breadth and depth while keeping the dataset manageable for thorough qualitative analysis. We acknowledge this cutoff may have excluded some relevant studies, but the subsequent expansion through Connected Papers was designed to mitigate this limitation. While no universally standardized number exists in the field of scoping reviews, this approach balanced representativeness with practicality. Articles were selected only if they addressed neurodegenerative conditions with high depressive comorbidity, as identified in the preselection phase. This process yielded a total of 147 articles.

### 2.4. Data Processing and Expansion

The next stage utilized “Connected Papers | Find and explore academic papers” [42] to expand the dataset. Connected Papers employs a sophisticated algorithm that analyzes approximately 50,000 articles for each query to select the few dozen with the strongest connections to the source article. Unlike traditional citation trees, Connected Papers arranges articles based on similarity metrics derived from co-citation and bibliographic coupling principles, meaning that papers with highly overlapping citations and references are presumed more likely to address related topics. The algorithm constructs a force-directed graph that visually clusters similar papers while distancing less similar ones from each other.

This approach allowed us to identify conceptually related studies even when they did not directly cite each other.

It is important to acknowledge certain limitations of this approach. First, the similarity metric relies heavily on citation patterns, which may disadvantage newer papers with fewer citations or papers from less-cited journals. Second, the algorithm’s dependence on the Semantic Scholar Paper Corpus means that coverage might vary across different scientific fields. Third, this methodology might potentially underrepresent paradigm-challenging articles that deliberately avoid citing mainstream literature. Despite these limitations, Connected Papers provided a valuable complementary approach to Elicit by identifying thematically related studies through bibliometric relationships rather than semantic content, thereby enhancing the comprehensiveness of our literature review.

When evaluating studies identified through Connected Papers, we applied specific criteria to assess their relevance and “closeness” to our research questions. First, conceptual relevance was prioritized over mere citation relationships; studies were evaluated based on their direct contribution to understanding the depression–neurodegeneration relationship, not just their bibliometric connection to seed papers. Second, we established a threshold of relevance where papers had to explicitly address at least one of our four hypothesized relationships to be included, rather than merely mentioning both depression and neurodegeneration in passing. Third, methodological rigor was considered when evaluating borderline cases, with preference given to studies employing longitudinal designs, adequate sample sizes, and validated assessment tools. During this expansion phase, our inclusion/exclusion criteria were applied slightly more liberally than in the initial selection to allow for the discovery of conceptually related work that might use different terminological frameworks or approach the relationship from novel angles. However, all studies ultimately included, in the final analysis, were subjected to the same strict final evaluation against our original inclusion criteria to maintain methodological consistency.

Inclusion and exclusion criteria were temporarily relaxed to facilitate broader exploration. The initial 147 articles were deduplicated to 131 and loaded into Connected Papers, generating a network of 40 closely related studies for each input. This process added 5240 articles to the dataset, which, combined with the original 131, resulted in a total of 5371 articles.

A second deduplication reduced the dataset to 3323 unique articles. Subsequently, titles and abstracts were analyzed, narrowing the selection to 216 studies. Full-text reviews of these 216 studies led to the final inclusion of 83 studies, guided by predefined inclusion and exclusion criteria.

Being a qualitative analysis of the existing literature, we only collected the relevant information of each included study in a text form, and divided them into the four existing theses highlighting the possible connections between neurodegeneration and depression, to then create a cohesive discussion. Therefore, we did not consider any specific study characteristic, but rather the content of each included entry.

Lastly, this scoping review was registered on the Open Science Framework (OSF) portal [43]. To consult the registered protocol, visit: https://doi.org/10.17605/OSF.IO/J2EAQ, accessed on 9 March 2025.

## 3. Results

**Figure 1:**
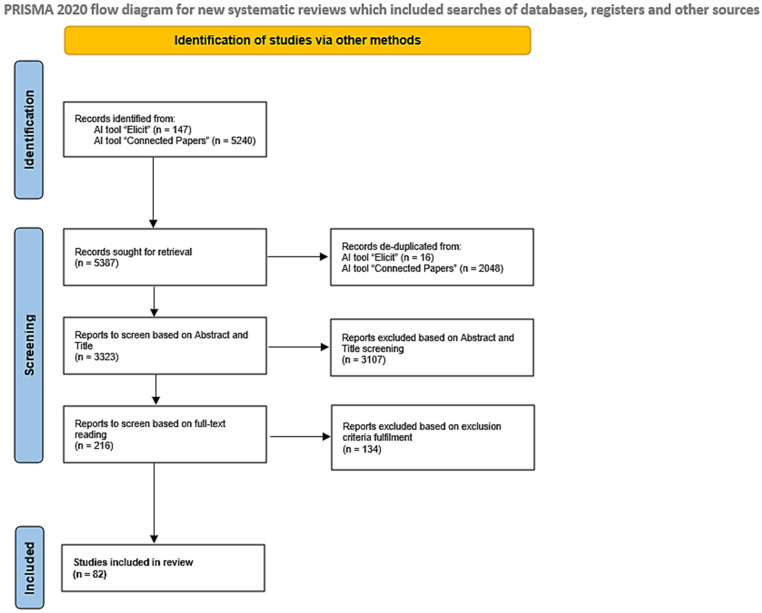
A flowchart illustrating the research strategy employed for this scoping review in accordance with PRISMA-ScR guidelines [44].

The identification process started on the 26th of July 2024 and ended on the 31st of August 2024. In total, through the double identification and selection process carried out with Elicit and Connected Papers, and based on the aforementioned exclusion and inclusion criteria, 82 citations (excluding citations regarding methods) were included in this scoping review. A detailed flow diagram illustrating the identification, screening, and inclusion steps is provided to offer a visual summary of the process (Figure 1).

The following provides a detailed analysis of the evidence collected based on each thesis undermining the relationship between the two clinically relevant conditions.

### 3.1. Depression as the Cause of Neurodegeneration

A retrospective cohort study showed that while depression diagnosed during the course of life predisposes to the development of dementia (especially VaD), on the other hand, depression diagnosed in old age predisposes the individual to AD. However, further studies would be needed to understand whether VaD might result from the triggering of degenerative processes caused by depression, while LLD may simply be a prodrome of AD [45,46]. Based on the literature, it is noteworthy how many studies compare depressive comorbidity between VaD and AD, revealing higher (and more severe) rates of depression in VaD compared to AD. It is remarkable to observe that this interpretation has not changed over the years [47,48,49,50,51,52,53,54,55,56,57,58]. In this regard, given that VaD seems to have an even higher rate of comorbidity with depression than AD, it is not difficult to think that depression could lead to the development of this form of dementia through other variables that mediate this interaction. These include “hypothalamic–pituitary–adrenal (HPA)” axis dysregulation leading to elevated cortisol levels that disrupt endothelial cell function, proinflammatory cytokines affecting blood–brain barrier permeability, and endothelial dysfunction—all of which create a pathophysiological bridge between depressive symptoms and cerebrovascular pathology [59]. An answer to this question could come from the proposal of [60], who labeled depression as a condition predisposing to a sort of “accelerated aging”, given that depressed individuals have a higher incidence of various aging-related diseases, including cardiovascular and cerebrovascular diseases. It may not be a coincidence that VaD falls squarely into this category.

However, although the heated debate between VaD and AD in terms of comorbid depression seems to favor VaD, the conclusions are not so different for AD either. A meta-analysis conducted by [61] concluded that a history of depression nearly doubles the risk of developing dementia in general, with particular reference to AD, while other studies have found a more than double [33,62] or even triple [63] risk increase. Additionally, a second meta-analysis showed that the odds ratios of developing AD in depressed individuals were positively related to the time interval between the diagnoses of depression and dementia across studies. Specifically, the authors argue that a positive relationship supports the hypothesis of depression being an independent risk factor rather than a prodrome. In contrast, if depression were a prodrome, the relationship between the interval and increased depression risk would be inverse, implying that depression would more likely occur closer to the time of dementia diagnosis [33,62].

On the other hand, consistent with the previously mentioned thesis that depression could be a prodrome of AD, other scientific evidence has confirmed this incidence for dementias in general, including MCI. Furthermore, refs. [64,65] observed that not only depression but also its severity was associated with an increased risk of MCI. Even meta-analyses, systematic reviews, and longitudinal studies, such as those by [66,67], and [68], respectively, concluded that depression, in addition to being a clear risk factor for MCI, also increases the likelihood of conversion to overt dementia.

A similar discussion appears to apply to PD, with numerous studies demonstrating the role of depression as a risk factor for the disease [69,70,71,72,73]. Among the most significant ones, a study conducted by [74] on a large cohort of young patients found that while only 259 out of 67,570 (0.38%) non-depressed patients developed PD, 19 out of 1358 (1.4%) depressed patients developed PD, showing a significant positive association between depression and subsequent incidence of PD. Specifically, individuals with depression were, on average, more than three times as likely to develop the disease compared to non-depressed subjects. Similarly, a large-scale study discovered that patients with depression were 3.24 times more likely to develop PD than the control patients [75], while a more recent meta-analysis concluded that depression was associated with a 2.20 times greater risk [76]. It is particularly noteworthy that, according to a systematic review and meta-analysis by [77], even early life stress experienced in the first years of life could cause depression through a dysfunctional adaptation of the HPA axis and mesocortical and mesolimbic dopaminergic pathways, which mediate mood, emotions, and/or cognitive functions, thereby predisposing the individual to an impairment of a neurophysiological basis common to both conditions, potentially laying the groundwork for the subsequent development of PD.

Remaining within the spectrum of DLB diseases, there is also evidence of a similar role for depression as a risk factor in other atypical parkinsonisms, including DLB [45]. It has even been observed that individuals with a history of depression have a six times greater risk of developing DLB [78]. One study, in particular, after surprisingly observing higher levels of α-synuclein in patients with depression, hypothesized that possible changes in the metabolism of this protein could be a biological factor in the transition from depression to DLB, concluding that depression is not only a prodromal symptom of DLB but also a causal risk factor for the pathology [79]. Along the same lines of research, another study observed that α-synuclein pathology is associated with depression, with a higher pathological load in the mesolimbic and nigrostriatal dopaminergic areas [80]. More recently, ref. [81] supported this thesis by demonstrating a lower density of dopaminergic neurons in the pars compacta of the substantia nigra in a sample of DLB patients with comorbid depression compared to non-depressed patients, highlighting an association between the aforementioned neuronal hypodensity and comorbid depression, and consequently, the role that this area may play as a mood modulation hub in patients with Lewy body spectrum diseases.

Finally, the hypothesis has also been raised that depression might be a risk factor for the development of ALS [82], but the evidence in the literature is not yet conclusive. It is also true that if dementia were truly a consequence or exacerbation of a depressive disorder, then depressed individuals with dementia who are treated with antidepressants should show some degree of slowed cognitive decline. Indeed, a study conducted by [83] demonstrated that treatment with antidepressants significantly reduced cognitive decline in depressed patients with AD.

However, as mentioned earlier, it is not possible to establish a clear cutoff that definitively determines how long before a dementia diagnosis one can talk about a “prodrome”, making it impossible to conclude with certainty whether depression is truly a risk factor for developing neurodegenerative diseases. For these reasons, some researchers have tried to support this hypothesis by exploring other avenues. One of these suggests that if depression were indeed a risk factor for the development of various forms of neurodegeneration, then it would be reasonable to expect that the likelihood of being diagnosed with dementia in old age depends on the recurrence of depressive episodes over a lifetime, as well as the severity of those episodes. Through a longitudinal study with a 17-year follow-up, ref. [84] demonstrated that depression increases the likelihood of developing dementia by 70%, and more specifically, ref. [85] confirmed through a multicenter study that the severity of depressive disorder was positively correlated with the likelihood of developing dementia. At the same time, ref. [86] found that the recurrence of depressive episodes significantly increased the risk of dementia. Additionally, the number of depressive symptoms also seems to be positively correlated with the likelihood of developing AD, with a 20% increased risk for each additional symptom [87].

Similar results have also been observed in relation to PD. In this case, it has been found that greater severity of depressive episodes significantly increases the risk of developing the disease. For example, in one study, a dose–response relationship was observed with the severity of depression, where patients admitted to hospital for depression had a significantly higher risk of developing PD than those receiving only outpatient care (OR 3.5, 95% CI 2.9–4.1), with the risk further increasing with the number of hospital admissions. This dose–response relationship strengthens the hypothesis of a potential causal link between depression and neurodegenerative processes [71]. These findings were further confirmed in a retrospective cohort study conducted by [88], which investigated the relationship between dysthymia depression, and dementia. The study found that depression indeed carries a greater risk of developing dementia compared to dysthymia, confirming the hypothesis that the severity of depression is an important variable to consider. This supports the notion of a “dose-effect” phenomenon, where the severity of depression (in terms of the number of episodes, the number of symptoms exhibited, and their severity) is proportional to the risk of developing some form of dementia during aging.

More recently, further research has sought to formulate alternative hypotheses to mechanistically explain these results, suggesting that depression might burden cognitive reserve, increasing the risk of dementia. For example, ref. [89] discovered that depression and cognitive reserve were closely related to cognitive performance and that low levels of cognitive reserve represented a vulnerability factor for unfavorable cognitive prognosis. Confirming this, ref. [90] found that depression and low cognitive reserve significantly increased the risk of subsequent dementia, and that higher cognitive reserve attenuated the risk associated with depression for developing dementia. According to these observations, if cognitive reserve indeed represents a defense against neurodegeneration, depression could be both an independent risk factor and an important mediator that, by altering cognitive reserve, simply unmasks a pathology that would manifest anyway, though with delayed onset [34]. Further evidence of the role that depression might play as a potential cause or risk factor for triggering neurodegenerative processes and/or for the development of dementia also comes from neurobiology. For years, the monoaminergic hypothesis has been the most explored, as the alterations found in the monoaminergic neurotransmitter systems responsible for the release of serotonin, norepinephrine, and dopamine underlie depression.

Confirming this, pharmacological treatments with antidepressants seem to regulate these neurotransmission systems. At the same time, monoaminergic neurons in the brainstem nuclei are also involved in the accumulation of both Aβ and α-synuclein, the main causes of two of the most widespread neurodegenerative diseases, namely AD and PD, as well as similar or prodromal conditions such as MCI [91]. However, over the years, many other neurobiological pathways have been identified that could provide further insights; in particular, some reviews, such as those by [30,60], have investigated various neuropathological pathways that concretely explain how depression could be a predisposing factor for neurodegenerative phenomena. Among these, one of the most well-documented findings has been the observation of reduced neuronal density in specific brain areas such as the prefrontal cortex, orbitofrontal cortex, anterior cingulate cortex, hippocampus, and amygdala in subjects with depression compared to healthy controls. Particular attention has also been paid to the role of neurotrophins, primarily the “brain-derived neurotrophic factor (BDNF)”. According to the neurotrophic hypothesis of depression, low levels of BDNF could reduce neurogenesis and lead neurons to cell death. Since this factor is essential for neuronal survival, its deficiency could promote the onset of neurodegenerative processes.

Moreover, another system that could act as a “bridge” between depression and neurodegeneration is the glial system. In this regard, a decrease in glial cells, which are crucial for the proper functioning and survival of neurons, has been observed in depressed individuals. Glial cells perform numerous essential functions, including regulating the extracellular environment and providing important nutrients. Furthermore, reduced levels of glial cells (especially astrocytes) alter glutamate signaling, causing an increase in glutamate along with a decrease in GABA, leading to excitotoxicity in cells. This abnormality would inevitably trigger a cascade mechanism capable of interfering with oxidative phosphorylation processes, generating an increase in oxidative stress, which in turn causes mitochondrial dysfunction. Recent findings also suggest that sleep disturbances, particularly short sleep duration, may contribute to cognitive decline through inflammatory pathways [92], further reinforcing the role of neuroinflammation in the depression-neurodegeneration link. Compromised oxidative phosphorylation can increase electron loss, which, reacting with molecular oxygen, produces the so-called superoxide anions, “reactive oxygen species (ROS)” that can damage DNA, proteins, and lipids, altering both their structure and specific function. This process occurs in parallel with the release of inflammatory cytokines and leads to nerve cell damage to the point of causing apoptosis, or cell destruction. This, in turn, further impairs neurotransmission, worsening the initial condition in a continuous and unstoppable loop mechanism.

It is also important to highlight a significant aspect; as mentioned earlier, these changes seem to occur within specific brain areas such as the prefrontal cortex, orbitofrontal cortex, anterior cingulate cortex, hippocampus, and amygdala, which are notably implicated in both cognition and emotional regulation, domains that are often altered in patients with both depression and neurodegenerative diseases. This finding is further supported by the discovery of neuropathological changes typical of some dementing forms, such as Lewy bodies, neurofibrillary tangles of hyperphosphorylated tau protein, and neuritic plaques of Aβ in depression [30,33,60,93,94,95]. Moreover, this hypothesis, which centers on microglia, becomes even more coherent when considering PD. Although microglia are present in many areas of the brain, they are particularly abundant in the pars compacta of the substantia nigra, where their alteration, along with the consequent release of proinflammatory cytokines and ROS, could explain why this area is most affected by the neurodegenerative process in PD and various parkinsonisms [71]. Furthermore, considering that oxidative stress is a central and characteristic phenomenon of many neurodegenerative diseases, one can understand how the link between depression and neurodegeneration is actually stronger than it might seem. It cannot be ruled out, in fact, that the onset of neurodegenerative processes could represent only the final stage of a path that began long ago, favored by a multiplicity of mechanisms, perhaps reversible and identifiable along a continuum, that ultimately lead to irreversible neurodegeneration. Additionally, closely related to the biochemical pathways mentioned is the functioning of the HPA axis, whose dysfunction is generally associated with stress, not only physical but also psychological (coping styles, social support, etc.), as well as numerous other genetic and epigenetic factors [60]. It has been widely demonstrated that HPA system dysfunction is a common feature among people suffering from depression. In fact, the majority of patients with severe depression have an altered glucocorticoid feedback functioning, which can cause an increase in cortisol levels in the body through a cascade mechanism [96]. In depression, this phenomenon, also known as hypercortisolemia, alters the function of glucocorticoid receptors, reducing it. Some antidepressants seem to have direct effects on these receptors, enhancing their functionality and increasing their expression [97,98]. At the same time, ref. [99] discovered that depressed patients who exhibit glucocorticoid resistance might be more vulnerable to developing neurodegenerative changes in the future due to chronic inflammatory processes that develop over time. Essentially, this means that chronic inflammation and HPA system dysfunction could contribute to the increased vulnerability of depressed patients to developing neurological problems, including neurodegenerative forms.

### 3.2. Depression as a Prodrome of Neurodegenerative Pathologies

Although depression may be a potential risk factor for the development of dementia, depressive symptoms should be considered from a different perspective. As previously mentioned, these symptoms could be viewed as a prodrome, a warning sign, indicating the onset of a form of dementia that has already been triggered by underlying neurodegenerative processes, which have not yet fully manifested. A widely discussed clinical entity that could be extremely useful in explaining the high comorbidity rate between depression and neurodegenerative diseases is the so-called “depressive pseudodementia”. In this regard, it is well known that depression is accompanied by significant cognitive symptoms, which makes it particularly difficult to distinguish between depressive and dementia-related symptoms, especially in elderly patients, due to the symptomatic overlap between the two conditions. In this context, “depressive pseudodementia” has been defined as a condition where an individual experiences reversible cognitive impairment secondary to a mood disorder, from which this condition presumably arises. Notably, pseudodementia has often been used to explain the manifestation of depressive symptoms as a prodrome of dementia rather than as a consequence of it. If pseudodementia manifests following a depressive disorder and, from a phenomenological standpoint, resembles dementia much more than the depression itself, it raises the question of whether, when depression appears later in life alongside cognitive decline, it might merely be a precursor, signaling the future onset of underlying dementia already in progress [60,100,101]. In other words, depression, with all its cognitive symptoms, could be the initial response to early neuroanatomical changes, which, as they progress, would increasingly worsen the individual’s cognitive profile, ultimately leading to full-blown dementia. Moreover, considering that the concept of “reversibility” inherent in pseudodementia has been increasingly questioned over the years—given that cognitive symptoms have been found to persist even after the remission of depressive symptoms—this argument takes on even greater theoretical significance. Supporting this, studies have shown that depressed individuals who exhibit cognitive decline are more likely to develop dementia than those with depression who do not show cognitive symptoms [32]. Thus, if this relationship were to be confirmed, it would provide an explanation for the high rates of comorbidity.

Moreover, complementary to the previously mentioned studies by [33,62], ref. [36] have been particularly illuminating in this regard, demonstrating that for every additional year between the onset of depression and the onset of dementia, the likelihood of developing dementia decreased by 8.4%. Furthermore, the twin analyses showed that those with a history of depression were three times more likely to develop dementia compared to their non-depressed twin counterparts, with a similar age of depression gradient. These results suggest that, after partially controlling for genetic influences, late-onset depression may be an early indicator of dementia rather than a direct risk factor.

Similarly, a longitudinal cohort study by [102] examined the relationship between depressive symptoms and dementia over a 28-year period, focusing on how these symptoms manifest at different stages of life. The results showed that depressive symptoms primarily appear in old age, about a decade before a dementia diagnosis, while there was no significant difference in depressive symptoms between those who developed dementia and those who did not until 12–28 years before the diagnosis. These findings are consistent with the hypothesis that LLD is not merely a long-term risk factor but rather a prodrome. Depression appears mainly in the preclinical phase of dementia, shortly before it is diagnosed, indicating that it could be an early manifestation of the underlying pathology rather than a separate cause that increases the risk of dementia in the future. Conversely, another longitudinal study by [103] reached similar conclusions despite obtaining opposite results, finding that while men with a history of depression, both past and present, had a significantly higher risk of developing dementia during the follow-up period, this increased risk was particularly evident only in the first five years of follow-up. Moreover, the use of antidepressants did not reduce this risk, leading the authors to conclude that depression could not be a risk factor but rather a prodrome.

However, as discussed earlier, when investigating the role that depression might play as a prodrome of dementia, it is important to select only studies that have specifically recruited subjects with LLD, excluding those where depression was diagnosed earlier in life, which are more likely to explain depression as a potentially causal risk factor. A thorough review by [32] revealed that compared to patients with early-onset depression, those with LLD are not only more likely to experience cognitive decline (compatible with the concept of pseudodementia) but also to develop true dementia over time. Additionally, from an anatomical perspective, patients with LLD are more likely to show both cortical atrophy and deep white matter lesions. It is still unclear whether LLD could be a prodrome for a particular type of dementia, but epidemiologically, it has been observed that subjects with LLD tend to develop AD or VaD, possibly because these are simply the most common forms of dementia. In fact, this trend has also been confirmed for PD by a recent systematic review and meta-analysis conducted by [104], which found that the subsequent risk of PD was significantly higher in patients with prodromal depression than in healthy individuals. Moreover, in the specific case of PD, the idea that depression might be a prodrome is perfectly consistent with [105]’s historical hypothesis, which first defined the staging of the disease. According to their observations, since the serotonergic nuclei of the Raphe (mainly involved in affective symptoms) undergo neurodegeneration starting from stage 2 when the substantia nigra’s pars compacta have not yet been affected by the neurodegenerative process (which begins to neurodegenerate from stage 3 of the disease), it is reasonable to think that depression could merely be a prodrome of the disorder [71].

### 3.3. Depression as a Consequence of Neurodegeneration

The evidence considered so far has focused on studies investigating depression as a potential risk factor or prodrome for neurodegenerative disease, dementia, or cognitive decline. In the previously mentioned studies, depressive disorder has always preceded the onset of dementia, albeit with varying intervals. Many of these are longitudinal studies that have observed how depression diagnosed earlier in life is associated with the subsequent development of a form of neurodegeneration. However, as initially mentioned, the relationship between these two conditions could be bidirectional, meaning that dementia itself might also cause depression. Regarding AD, this proportion significantly increases within five years, with as many as three out of four dementia patients developing a depressive disorder. In fact, when considering specific forms of dementia, such as VaD, FTD, and DLB, the estimates may be even higher [106]. For instance, depression is known to be a frequent complication occurring in 25–80% of patients with VaD during the course of their illness [107], suggesting that the latter may predispose, perpetuate, or precipitate into depressive syndromes [108].

It may seem somewhat obvious to emphasize that, particularly in the early stages of the disease, the awareness of a grim diagnosis, the limitation of one’s cognitive and sometimes even motor independence (e.g., parkinsonism), combined with the bleak outlook on life, can significantly dampen a patient’s mood, leading to an emotional reaction that drives them (and their family members) into a deep depressive state [105]. However, while it may seem logical to consider depression as the result of a psychological breakdown during the initial phase of acceptance, it is crucial to note that the neuroanatomical changes associated with a degenerative disease can affect areas involved in emotional processing. It is now clear that many studies have suggested a correlation between dementia and depression in the elderly. This correlation could be due to similar structural correlates, including white matter lesions and cortical atrophy. Although no study has yet examined whether these lesions appear before dementia and depression independently of each other in the general population, a 10-year longitudinal study clarified this aspect, discovering that both white matter lesions and temporal lobe lesions predicted the onset of dementia independently of depression and vice versa, meaning the onset of depression even after controlling for dementia [109]. A review by [39] also concluded that, although depression in the early stages of AD might arise as a reactive psychological response to adaptation difficulties, in the later stages, it would indeed be the result of cognitive deterioration in brain areas involved in emotional responses.

Furthermore, as one might expect, MCI has also been associated with an increased risk of developing depression, as demonstrated by a longitudinal study by [110]. However, it is even more significant to highlight that depressive symptoms in MCI increase the risk of progressing to AD [111].

For common disorders within the Lewy body spectrum, such as PD with and without dementia and DLB, there is evidence that degeneration of the dopaminergic circuit can influence depressive symptoms in these disorders, given that this neurotransmitter is primarily involved in the reward and motivation circuit [80]. In fact, in PD, it seems that an altered metabolism of anatomical systems responsible for serotonin production, known as the “mood neurotransmitter”, might play a crucial role as a predisposing factor for the development of depression [112]. Moreover, dysfunctional dopaminergic and serotonergic systems have also been observed in FTD [113].

Conversely, there has been no strong evidence found for other atypical parkinsonisms, with the exception of a particularly intriguing single-case study involving CBD. In this study, researchers examined a 60-year-old woman with a long history of depression treated with antidepressants. Despite extensive antidepressant therapy, which was even increased in the two years before hospitalization, the patient experienced worsening depressive symptoms and the development of neurological disorders that, following a Magnetic Resonance Imaging (MRI), revealed brain atrophy, leading to the diagnosis of CBD. The researchers found that both neurological and depressive symptoms improved with the administration of levodopa, suggesting that the recurrent depression might have been induced by CBD [114].

However, in the specific case of ALS, the evidence is controversial, with depression potentially manifesting either before or after the ALS diagnosis [17]. However, in the latter scenario, it is thought that depression might represent only a reactive response to the diagnosis, given the decrease in symptoms over time [115]. Nevertheless, a study by [17] conducted on 1752 patients investigated this comorbidity by following the patients both before and after the ALS diagnosis. It was observed that ALS patients have a higher risk of being diagnosed with depression both before and after the grim diagnosis. However, the important data to note is that if they had a higher risk of receiving a clinical diagnosis of depression compared to controls before the ALS diagnosis, this risk would be increased in the year preceding the ALS diagnosis and become even higher one year after the diagnosis. This increase suggests that depression might be more of a consequence (or at least a prodrome) of ALS than a risk factor: a prodrome because it cannot be ruled out that the early symptoms of ALS (which may not yet be recognized as such) are already triggering a neural substrate, leading to the manifestation of depression; a consequence because if depression were primarily a risk factor for ALS, one would expect elevated depression rates well before the diagnosis, not just in the year before or after the diagnosis. The timeliness of the diagnosis must also be considered. In this regard, a study by [116] clearly showed that the length of the diagnostic interval within which the diagnosis occurred predicted depressive symptoms, highlighting the role that a late diagnosis can have in the development of reactive depression.

These findings are of immense importance because they demonstrate that depression and dementia might have common neuropathological bases. Considering the structures involved in the medial temporal lobe, it is not improbable that, just as depression might contribute to the onset of dementia, dementia might also impair the same structures involved in emotion regulation, causing depression. Following this line of reasoning, it might better explain why it is still unclear whether the use of antidepressants can be an effective aid to therapy, with studies reaching conflicting conclusions on their real efficacy [117,118]. On the one hand, the use of antidepressants might enhance neurotransmission in areas common to both conditions. On the other hand, the neurodegeneration of areas involved in emotional regulation might negate their effect.

### 3.4. Depression and Neurodegeneration as a Spurious or Dissociated Relationship

In conclusion, as mentioned initially, a final perspective considers depression and neurodegenerative phenomena as distinct components, whose pathological manifestations might depend on a third variable that links the two conditions. This could include factors such as an underlying medical condition, low socio-economic status, education level, etc. [119,120].

Moreover, despite studies not pointing in this direction, it cannot be ruled out that depression and neurodegenerative diseases might actually be entirely separate and independent conditions. From an epidemiological standpoint, with the significant increase in dementia cases observed over the past century, alongside depression—which is expected to become the leading global disease by 2030 [121]—we cannot exclude the possibility that, from a purely statistical perspective, there might be a large segment of the population where these two conditions coincidentally occur in comorbidity.

### 3.5. Limitations

This scoping review is not without limitations that warrant discussion.

A significant methodological limitation concerns the uneven coverage of the four hypotheses presented in this review. It is important to acknowledge that the evidence supporting a spurious or dissociated relationship between depression and neurodegeneration is notably less robust than for the other hypotheses examined in this review. The brevity of this section reflects a significant gap in the literature rather than a lesser importance of this perspective. Few studies have systematically examined the possibility that the observed comorbidity might be coincidental or mediated entirely by third variables, despite the epidemiological plausibility of such an explanation, given the high prevalence of both conditions in aging populations. This limitation highlights an important area for future research, as rigorously designed studies specifically testing null or spurious association hypotheses could provide valuable insights and potentially challenge current assumptions about the nature of the relationship between depression and neurodegeneration. The lack of comprehensive evidence in this area should be considered when interpreting the overall findings of this review.

One major issue concerns the use of artificial intelligence-based tools, such as Elicit and Connected Papers. While these tools facilitated the identification and expansion of the dataset, they introduced potential biases inherent in their automated article selection processes. For example, Connected Papers generates networks of related articles that vary with each iteration of the same input, which undermines the ability to establish a fully replicable search methodology. Additionally, Elicit tends to prioritize articles from highly visible journals, potentially excluding relevant studies published in less prominent venues.

It is important to explicitly acknowledge how these AI-based methodological choices may have influenced our results and conclusions. First, Elicit’s semantic analysis algorithms might have favored certain interpretations of the depression–neurodegeneration relationship based on the predominant language patterns in scholarly literature, potentially amplifying existing biases in the field. For instance, the significantly larger section on depression as a risk factor compared to other hypotheses might partially reflect Elicit’s tendency to prioritize well-established research trajectories with more standardized terminology. Second, Connected Papers’ reliance on citation patterns could have created echo chambers where certain theoretical perspectives are overrepresented due to high rates of self-citation within research communities. This may have underrepresented emerging or less mainstream perspectives, particularly regarding the hypothesis of spurious relationships between depression and neurodegeneration. Third, both tools may have systematically underrepresented studies from non-English speaking countries or from disciplines that use different terminological frameworks, potentially limiting the cultural and disciplinary diversity of perspectives included. Collectively, these AI-related biases may have created a somewhat skewed representation of the evidence base, potentially overemphasizing certain mechanistic explanations while underrepresenting others, despite our efforts to maintain analytical balance throughout the review process.

Consequently, the findings should be interpreted with caution, as some studies may contain inherent limitations.

It should be noted that the section examining depression as a cause of neurodegeneration is more extensive than other sections. This reflects the current state of the literature, where this hypothesis has received the most substantial research attention. The greater volume of studies exploring depression as a risk factor or causal agent necessitated a more detailed analysis to adequately capture the diverse mechanisms proposed and the evidence presented. While this might appear to suggest a bias toward this hypothesis, we have made every effort to provide balanced evaluations of all four potential relationships. The proportional length of each section is intended to reflect the current distribution of research attention within the field rather than suggesting a bias toward any specific hypothesis. Future studies that delve deeper into alternative relationships may ultimately result in more equitable section lengths in future reviews.

Furthermore, no temporal filter was applied during article selection. While this allowed for the inclusion of a broad range of studies, some older articles may not reflect current knowledge and recent advancements in understanding the relationship between depression and neurodegeneration. This lack of temporal filtering also represents a missed opportunity to conduct a formal analysis of how research trends, methodologies, and conclusions regarding the depression–neurodegeneration relationship have evolved over time. While our primary objective was to provide a comprehensive overview of the existing evidence regardless of publication date, thus capturing the full spectrum of research contributions to this field, a temporal trend analysis could have revealed important shifts in theoretical frameworks, improvements in methodological approaches, or changes in the understanding of underlying mechanisms across decades. For instance, earlier studies might have focused more on phenomenological observations, while recent research increasingly incorporates advanced neuroimaging, genetic analyses, and biomarker studies. Additionally, the conceptualization of both depression and neurodegenerative disorders has evolved substantially over the years, with diagnostic criteria undergoing significant revisions. Without systematic temporal analysis, our review may not adequately capture these evolutionary trends in the field, potentially obscuring important insights about the progressive refinement of our understanding of these complex relationships. Future reviews could address this limitation by specifically analyzing how evidence for each hypothesis has developed chronologically and how methodological advancements have influenced research conclusions.

Far from being a compromise, the decision to use AI tools reflects a conscious effort to enhance the efficiency and depth of the review process. By leveraging AI, this scoping review was able to uncover patterns and connections within the literature that might have been missed through manual methods alone, providing an initial overview that can inform future in-depth investigations. Previous studies have successfully employed these tools to navigate large datasets, demonstrating their value in improving efficiency and uncovering connections that might otherwise be overlooked. This scoping review builds upon these advancements, leveraging AI to manage the complexity of the literature in an innovative way. In this regard, ref. [122] emphasizes the role of AI in making a more efficient and systematic reviewing process. The adoption of AI tools for scientific research is increasingly supported by studies such as [123,124], which highlight how AI-based tools can simplify the process of identifying and synthesizing scientific evidence, improving both accuracy and efficiency to the point of achieving levels comparable to those of human experts. Furthermore, this approach not only significantly accelerates article selection while maintaining the same methodological rigor but also reduces the risk of human error [125,126]. The exclusive use of these tools, while not totally eliminating the risk of bias, allowed for the structured and transparent selection of a vast corpus of articles based on predefined criteria. Elicit’s ability to generate results based on specific questions and assess the significance of academic citations, combined with Connected Papers’ functionality for identifying conceptual links between articles, enabled a more in-depth review compared to manual methods.

Considering everything, it is worth emphasizing once again that the methodological choices made in this scoping review reflect the constraints and priorities associated with the scope and objectives of the study. As mentioned, these decisions were guided by the exploratory nature of this work, which prioritized mapping a broad range of evidence to identify patterns and trends through a broad overview rather than providing a narrow synthesis or definitive conclusions.

Future studies with more focused objectives or resources could address these limitations through a more rigid and predefined framework.

## 4. Discussion

In this review, we summarize the evidence supporting each of the initial hypotheses on the relationship between depression and the development of neurodegenerative diseases, attempting to define a clear directionality. Among these, the discussion included depression as a risk factor or, more incisively, as a potential cause of neurodegeneration; depression as a prodrome; depression as a consequence; and depression and neurodegeneration as events that are either spuriously linked or randomly co-occurring in the population. It is certainly possible that these different theories could partially coexist without excluding one another. As we discussed, it is likely that the neuropathological mechanisms associated with depression may increase an individual’s susceptibility to neurodegeneration, especially when it occurs at an early stage. At the same time, it is not impossible that when neurodegeneration emerges only in old age, it could merely represent a prodrome, a mere reflection of an already initiated latent neurodegenerative process that will only surface later. Similarly, if depressive symptoms appear following a diagnosis of dementia, they could simply be a psychological symptom of an ongoing anatomical change. Finally, it must also be considered that there may be no direct causal relationship between the two conditions, but rather a common overlap of variables that independently cause the onset of both pathologies—or that they may not actually be associated at all.

It is also important to acknowledge that our review has uncovered several noteworthy contradictions in the existing literature. First, while numerous longitudinal studies suggest that depression increases the risk of subsequent neurodegeneration, the temporal association varies significantly across studies, with some finding stronger associations for early-life depression and others for late-life depression. Second, the neurobiological mechanisms proposed to explain how depression might lead to neurodegeneration (such as HPA axis dysregulation, inflammation, and oxidative stress) are the same processes implicated when examining neurodegeneration as a cause of depression, creating circular explanations in the literature. Third, studies investigating depression as a prodrome versus a risk factor often use similar methodological approaches but reach different conclusions based on how they interpret the temporal relationship between depressive symptoms and cognitive decline. Fourth, the evidence regarding treatment effects is particularly inconsistent, with some studies suggesting that treating depression mitigates cognitive decline while others find no protective effect despite improvement in mood symptoms. These contradictions likely stem from the heterogeneity of both depression and neurodegenerative conditions, methodological differences across studies, varying definitions of key constructs, and the inherent challenge of establishing causality in complex, multifactorial conditions that develop over decades. Rather than viewing these contradictions as limitations, we suggest they reflect the true complexity of the relationship and highlight the need for more nuanced research paradigms.

Despite the extensive literature reviewed, establishing a definitive causal direction between depression and neurodegeneration remains challenging. While each hypothesis has supporting evidence, our review highlights the likely bidirectional nature of this relationship, with multiple interaction points at biological, psychological, and environmental levels. Rather than viewing these hypotheses as competing explanations, they may represent different aspects of the same complex phenomenon, operating simultaneously or sequentially depending on individual factors and disease trajectory.

At present, while the hypothesis of depression as a risk factor has been the most thoroughly explored in the literature [127], our review suggests that no single hypothesis can fully explain the complex relationship between depression and neurodegeneration. Rather, the evidence points toward a nuanced, context-dependent interaction where multiple mechanisms may operate simultaneously or sequentially depending on individual factors, disease type, and stage of progression. The extensive research on depression as a risk factor reflects the historical focus of the field but does not necessarily indicate stronger empirical support for this hypothesis over others.

**Figure 2:**
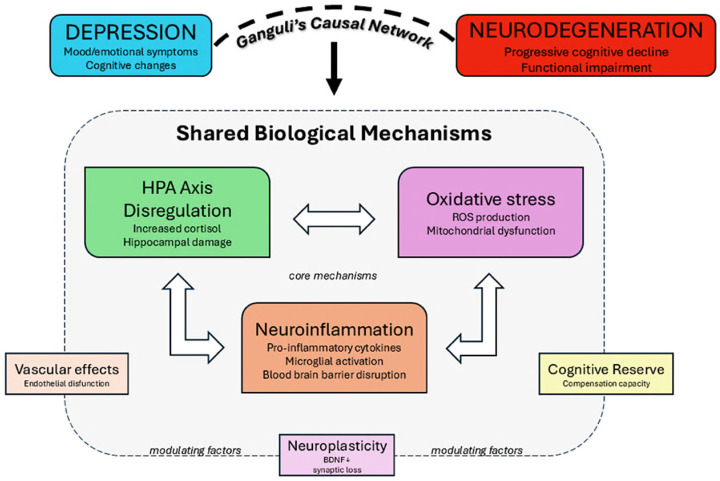
Ganguli’s causal network model illustrates the shared biological mechanisms and bidirectional pathways connecting depression and neurodegeneration.

To address the questions surrounding the nature of the link between depression and neurodegeneration, Ganguli [128] proposed the concept of a “causal network”, emphasizing that this relationship is likely so complex that it cannot be explained in purely dichotomous terms. Instead, it calls for a consideration of a series of variables that, by interacting with one another, contribute to a syndromic picture in which depression and neurodegeneration are just the tip of the iceberg. As Ganguli suggests, these interacting variables include biological factors (HPA axis dysregulation leading to sustained hypercortisolemia and hippocampal damage), psychological factors (reactions to cognitive decline awareness), vascular pathology (cerebrovascular disease affecting prefrontal systems that mediate both mood and executive functions), cognitive reserve (education and premorbid intellectual functioning), and the pathological processes underlying specific neurodegenerative diseases. These factors may create multiple bidirectional pathways where depression could be a risk factor, prodrome, consequence, or independent co-occurring condition with neurodegenerative diseases (Figure 2).

**Figure 3:**
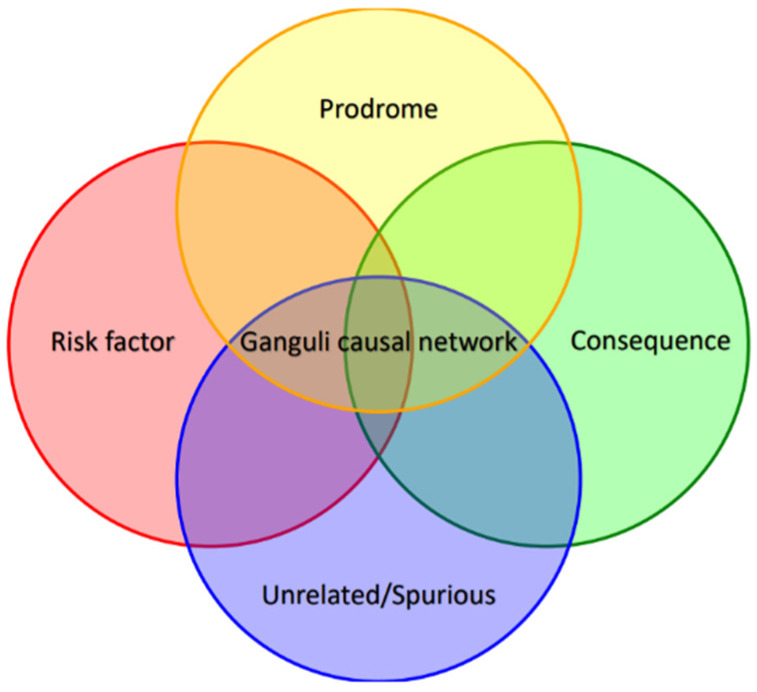
A graphical representation of the four trajectories influenced by possible common variables. The diagram illustrates how these trajectories may coexist and interact rather than being mutually exclusive.

It is important to accept that, to date, this view likely remains the best explanation for the complex relationship between these conditions. In other words, the connection between depression and neurodegeneration, if it exists, is much more complex than one might imagine, as the constructs and hypotheses involved could be just a drop in the ocean of variables that come into play in mediating the relationship between two conditions that, to this day, remain largely unknown (Figure 3). For this reason, despite the undeniable theoretical contribution that this review adds to the literature, this line of research remains far from conclusive.

The findings of this review have significant implications for psychiatry, neurology, and public health. From a nosological perspective, the complex bidirectional relationship between depression and neurodegeneration challenges current categorical diagnostic systems that often treat these conditions as distinct entities. This suggests that more dimensional and integrated classification approaches may better capture their overlapping neurobiological substrates and clinical presentations. Clinically, greater knowledge could improve the ability to identify at-risk patients early on, enabling timely interventions that could slow or alter the course of neurodegenerative diseases. Treatment strategies should be tailored based on the temporal relationship between depressive and neurodegenerative symptoms: when depression appears to be a risk factor, aggressive and sustained treatment alongside cognitive enhancement strategies may be warranted; for prodromal presentations, closer monitoring for both conditions might preserve cognitive function longer; when depression emerges as a consequence, approaches should address both neurochemical imbalances and psychological adaptation to cognitive decline.

From a research perspective, clarifying this relationship offers new insights into pathogenic mechanisms, supporting the development of targeted interventions such as early screening protocols, cognitive reserve enhancement strategies, neuroinflammation-targeting therapies, and approaches to HPA axis regulation. Future therapeutic directions could include dual-action compounds that simultaneously address both conditions, personalized pharmaceutical approaches based on inflammatory profiles, specialized cognitive-behavioral interventions, and lifestyle modification programs targeting common vascular risk factors. Additionally, identifying specific biomarkers that distinguish between depression as a risk factor versus a prodrome would allow stratification of patients into appropriate treatment pathways. Longitudinal studies combining neuroimaging, genetic analysis, and detailed clinical phenotyping will be particularly valuable in establishing temporal dynamics and identifying critical windows for intervention. Finally, these advances could foster greater integration between mental health and neurological services, promoting a more holistic and personalized approach to patient care for these interrelated conditions.

## 5. Conclusions

This scoping review sheds light on the intricate interplay between depression and neurodegeneration, underscoring the several ways in which these conditions may influence each other’s progression. Our findings suggest that depression can be a risk factor, prodrome, and result of neurodegenerative diseases, especially Alzheimer’s disease, vascular dementia, and Parkinson’s disease. The evidence reveals a bidirectional relationship, suggesting that neurodegenerative processes can worsen depressive symptoms, while chronic depression may drive neurodegenerative changes through neurobiological mechanisms, including inflammation and dysregulation of the HPA axis. The implications for psychiatric classification are significant. Current diagnostic systems frequently categorize depression and neurodegeneration as separate conditions. However, our findings support a more cohesive approach that recognizes their shared neurobiological substrates. Clinically, this highlights a nuanced understanding of patient presentations, where early identification of depressive symptoms in at-risk populations could facilitate timely interventions, potentially altering the trajectory of neurodegenerative diseases. Treatment strategies should be tailored to the temporal relationship between depressive and neurodegenerative symptoms. For patients where depression is identified as a risk factor, proactive management of depressive symptoms may be crucial in delaying or preventing cognitive decline. Conversely, when depression arises as a consequence of neurodegeneration, therapeutic approaches should focus on both mood stabilization and cognitive support. Future research should prioritize longitudinal studies that explore the causal pathways linking these conditions, aiming to identify biomarkers that differentiate between depression as a risk factor versus a prodrome. Such insights could inform personalized treatment pathways, fostering a holistic approach to care that integrates mental health and neurological services.

## Abbreviations

The following abbreviations are used in this manuscript:

AD	Alzheimer’s disease
VaD	Vascular dementia
PD	Parkinson’s disease
DLB	Dementia with Lewy bodies
CBD	Corticobasal degeneration
PSP	Progressive supranuclear palsy
FTD	Frontotemporal dementia
ALS	Amyotrophic lateral sclerosis
MCI	Mild cognitive impairment
LLD	Late-life depression
PRISMA-ScR	PRISMA guidelines for scoping reviews
HPA	Hypothalamic–pituitary–adrenal
BDNF	Brain-derived neurotrophic factor
ROS	Reactive oxygen species
